# Steamed Ginseng Berry Powder Ameliorates Skeletal Muscle Atrophy via Myogenic Effects

**DOI:** 10.4014/jmb.2309.09017

**Published:** 2023-11-17

**Authors:** Jungbum Kim, Hui-Ji Choi, Donghyuk Seo, Sang-Ah Lee, Jong Beom Heo, Dong Hyuk Baek, Wonhwa Lee, Gyu Yong Song

**Affiliations:** 1 Department of Chemistry, Sungkyunkwan University, Suwon 16419, Republic of Korea; 2 College of Pharmacy, Chungnam National University, Daejeon 34134, Republic of Korea; 3 Faculty of Biotechnology, College of Applied Life Sciences, Jeju national University, Jeju 63243, Republic of Korea; 4 Environmental Safety Group, Korea Institute of Science and Technology (KIST) Europe, Saarbrücken 66123, Germany; 5 AREZ Co., Ltd., Daejeon 34036, Republic of Korea

**Keywords:** Sarcopenia, steamed ginseng berry powder, skeletal muscle atrophy, myogenesis

## Abstract

Sarcopenia is an age-related loss of muscle mass and function for which there is no approved pharmacological treatment. We tested direct efficacy by evaluating grip strength improvement in a sarcopenia mouse model rather than drug screening, which inhibits specific molecular mechanisms. Various physiological functions of ginseng berries are beneficial to the human body. The present study aimed to evaluate the efficacy and safety of steamed ginseng berry powder (SGBP). SGBP administration increased myotube diameter and suppressed the mRNA expression of sarcopenia-inducing molecules. SGBP also reduced the levels of inflammatory transcription factors and cytokines that are known to induce sarcopenia. Oral administration of SGBP improved muscle mass and physical performance in a mouse model of sarcopenia. In summary, our data suggest that SGBP is a novel therapeutic candidate for the amelioration of muscle weakness, including sarcopenia.

## Introduction

The skeletal muscle is the largest organ in the human body and is involved not only in locomotion but also in biological activities, such as energy expenditure, glycogen storage, and thermoregulation. Its malfunction causes various secondary diseases, adversely affecting the quality of life in old age [1, 2]. Muscle mass is determined by the catabolic–anabolic balance and fusion rate of muscle progenitor cells [3]. Many clinical conditions, such as lung disease, glucocorticoids, starvation, cancer, and aging, cause muscle atrophy because the rate of protein degradation is higher than that of protein synthesis [4-8]. This is attributed to the expression of ubiquitin ligases, atrogin-1, and muscle ring finger 1 (MuRF1). Silencing atrogin-1/MuRF1 in myoblasts inhibits the proteolysis of myoblast-determination protein 1 (MyoD), a transcription factor that regulates myogenic progenitor cell function and differentiation [9]. Expression of Foxo3a, a transcriptional activator of the atrogin-1/MAFbx gene, is elevated in the muscles of aged mice [10, 11]. Therefore, the atrogin-1/MuRF1 axis may serve as an effective target for preventing age-related muscle loss.

Skeletal myogenic differentiation is mediated by various muscle-regulatory factors (MRFs), such as MyoD, myocyte-enhancing factor 2 (MEF2), myogenin (MyoG), myogenic determination factor 5 (Myf5), and MRF4 [12, 13]. Muscle marker genes, such as myosin heavy chain (MyHC) and muscle creatine kinase (MCK), are substantially elevated in differentiated myotubes and regulate muscle activity [14, 15]. Skeletal muscle atrophy is induced by inflammatory cytokines, such as TNF-α, IL-1β, and IL-6 [16]. Previous studies have reported that inflammatory cytokines accumulate with age, causing a redox imbalance. Nutritional deficiencies and chronic inflammatory diseases are common conditions that contribute to the progression of inflammatory sarcopenia. Accelerated or exaggerated muscle loss increases the morbidity and mortality associated with chronic inflammatory diseases. Therefore, enhancement of myogenic differentiation could protect against injury-induced severe skeletal muscle loss and improve the management of inflammatory diseases.

Several studies of *Panax ginseng* have been conducted to determine the mechanisms of its efficacy and to elucidate anti-inflammatory actions after administration [17]. Ginsenosides, triterpene saponins, are a family of molecules uniquely found in *Panax ginseng* that commonly have a four-ring structure with various sugar groups (*e.g.*, glucose and rhamnose) attached to it [18]. Through a repeated process of steaming and drying, the sugar groups in ginsenosides are deglycosylated, converting them into an enhanced absorbable form, namely rare ginsenosides [19]. However, until now, only the root portion of *Panax ginseng* has been consumed while the remaining stem and berry parts have been discarded. In this regard, our research team determined the optimal steaming-included process conditions for ginseng berry extract, transforming the main ginsenosides into more effective and absorbed rare ginsenosides. Owing to its anti-inflammatory properties, this compound has the potential to be used as a health supplement or drug. The previously developed RGX365, manufactured by large-scale separation and purification of six Protopanaxatriol (PPT)-type rare ginsenosides, was used for comparison.

In the present study, the anti-inflammatory effects of steamed ginseng berry extract (SGBP) were demonstrated following in vivo administration in senile mice with sarcopenia. We observed improvements in muscle strength and total muscle mass, along with enlarged muscle fibers. In parallel, the expression of sarcopenia-inducing molecules and inflammatory response signals was downregulated. Additionally, toxicity tests of blood cytokine levels, blood cell damage, and organ damage were conducted for further application in humans.

## Materials and Methods

### Preparation of Steamed Ginseng Berry Extract (SGBP)

**Fig. 1:**
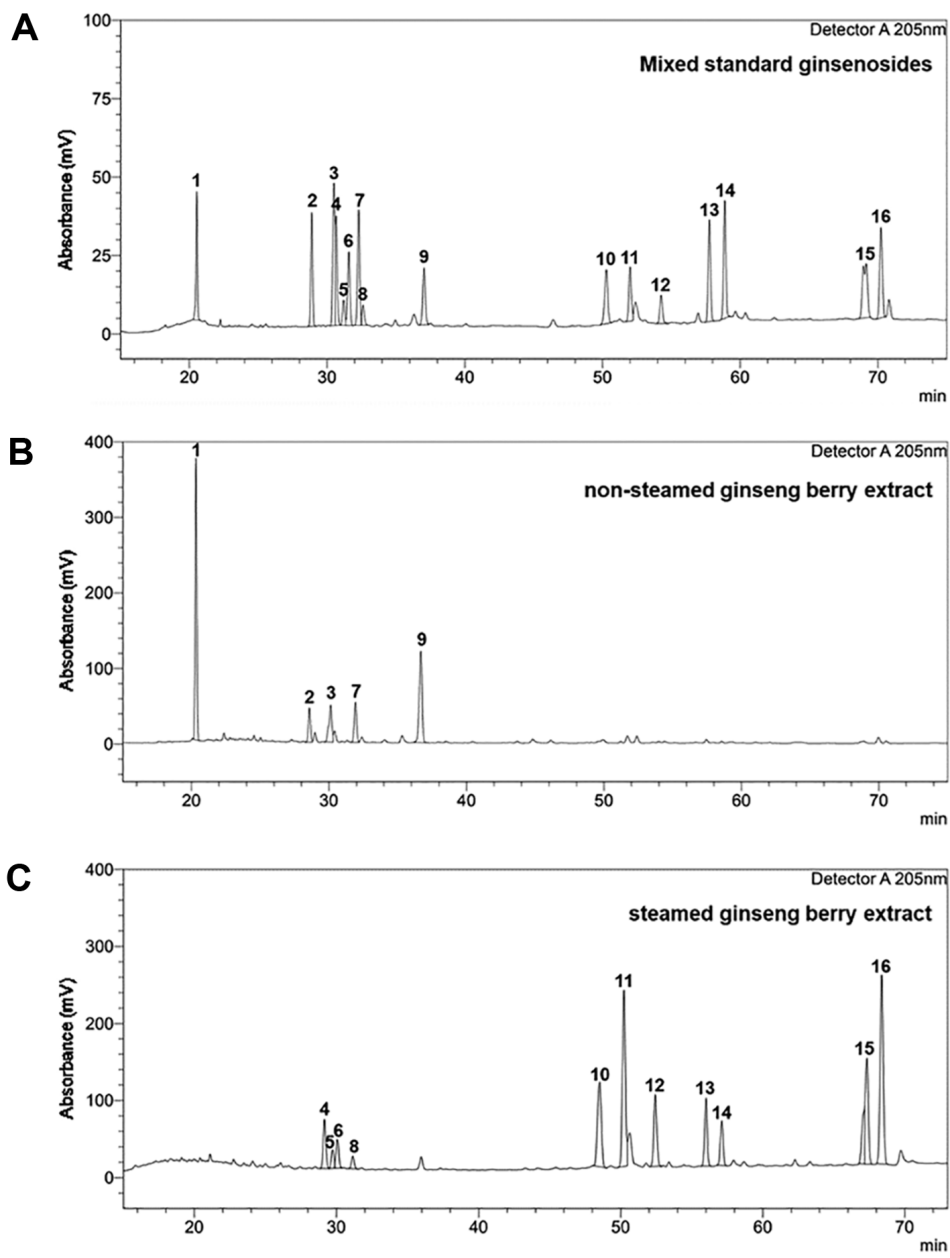
HPLC chromatograms for (A) mixed standard ginsenosides, (B) non-steamed ginseng berry extract, and (C) steamed ginseng berry extract. Each ginsenoside was analyzed using an HPLC system fitted with a C-18 column utilizing a solvent gradient system. The ginsenosides were identified by comparing the retention times with standard ginsenosides. Peaks: 1. Re; 2. Rb1; 3. Rc; 4. 20(S)-Rg2; 5. 20(S)-Rh1; 6. 20(R)-Rg2; 7. Rb2; 8. 20(R)-Rh1; 9. Rd; 10. Rg6; 11. Rg4; 12. Rh4; 13. 20(S)-Rg3; 14. 20(R)-Rg3; 15. Rk1; 16. Rg5.

Steamed ginseng berry extract containing PPT- and PPD-type rare ginsenosides (20(S)-Rg2, 20(S)-Rh1, 20(R)-Rg2, 20(R)-Rh1, Rg6, Rg4, Rh4, 20(S)-Rg3, 20(R)-Rg3, Rk1, and Rg5) was obtained from fresh ginseng berries. To elaborate, fresh ginseng berries (100 g) were washed with cold water and introduced into 1 L of 80% aqueous ethanol solution. The mixture was extracted at 70°C for 6 h and filtered; the same process was repeated three times. The resulting filtrates were combined and subjected to vacuum. Consequently, 60 g of steamed ginseng berry extract was obtained with a yield of 60%. Standards of PPD- and PPT-type main and rare ginsenosides were purchased from the Ambo Institute. Co., Ltd. (Korea). The content of each rare ginsenoside in the steamed ginseng berry extract was analyzed using high-pressure liquid chromatography (HPLC) (Fig. 1). HPLC analysis was carried out on Shimadzu Nexera series instrument with a UV detector and an ACE 5-C18 column (250 × 4.6 mm). The separation was achieved by gradient elution as follows: 0–10 min (20% B), 10–18 min (20–33% B), 18–40 min (33–40% B), 40–65 min (40–60% B), 65–75 min (60% B), 75–80 min (60–80% B), 80–85 min (80% B), 85–87 min (80–20% B), 87–95 min (20% B), A (H_2_O) and B (Acetonitrile). The solvent flow rate was held constant at 1 ml/min, and the sample injection volume was 20 μl. In steamed ginseng berry extract, the content of each rare ginsenoside 20(S)-Rg2, 20(S)-Rh1, 20(R)-Rg2, 20(R)-Rh1, Rg6, Rg4, Rh4, 20(S)-Rg3, 20(R)-Rg3, Rk1, and Rg5 was 5.01%, 1.86%, 2.99%, 1.37%, 12.84%, 20.56%, 8.41%, 7.11%, 4.63%, 12.15%, and 23.07%, respectively.

### Animal Experiments and Drug Preparation

C57BL/6 mice (2-month-old, male); (23-month-old, male) were obtained from Orient Bio (Republic of Korea). Animals were housed in polycarbonate cages under controlled temperatures at 20–25°C and humidity of 40–45%, with a 12:12 h light/dark cycle. The mice were fed a normal rodent pellet diet and provided with water ad libitum. Five animals were housed in each cage and used after a 12-day acclimatization period. All the animals were treated according to the guidelines of the Institutional Animal Care and Use Committee (IACUC) of Sungkyunkwan University (IACUC No.: 202111291).

The old mouse group (23-month-old) was fed steamed or non-steamed ginseng berry extract (SGBP, NSGBP) at different doses (25 or 50 mg/kg) or RGX365 (5 mg/kg) once daily for 30 days. SGBP, NSGBP, and the ginsenoside RGX365 were purchased from AREZ Co., Ltd. (Republic of Korea). The extract powder was dissolved in 100 μl solution and administered through oral gavage using a ball tip needle.

Following 30 days of oral administration, grip strength was measured using a grip strength test (Bioseb) before sacrifice. Grip strength was measured using standard protocols by pulling the tail of the mouse hanging on the apparatus until it released the grasping tool. After five measurements per mouse, the mean of each peak force was divided by body weight.

### Histological Staining

After euthanasia, the tibialis anterior (TA) muscle was exposed. The fascia was carefully peeled off with forceps and the TA muscle was separated. The TA muscle tissue was embedded in optimal cutting temperature (OCT) compound (Sakura Fintech) and snap-frozen in liquid nitrogen. Samples were cross-sectioned in 10 μm thickness at the midbelly region; hematoxylin and eosin (H&E) staining was performed using standard protocols.

### Immunohistochemistry and Morphometric Analysis

Other snap-frozen TA muscle sample slides (10 μm thickness) were used for immunohistochemistry analysis. The slides were fixed in 4% paraformaldehyde (PFA) or acetone, blocked in 2% bovine serum albumin (BSA) in phosphate-buffered saline (PBS) for 1 h following overnight incubation with mouse anti-MyHC (sc-32732, Santa Cruz Biotechnology USA), rat anti-laminin (MAB1914P, Millipore, USA), mouse anti-AREG (sc-74501, Santa Cruz Biotechnology), mouse anti-e-MyHC (sc-53091, Santa Cruz Biotechnology), rabbit anti-laminin (ab11575, abcam), and mouse anti-MyoD (sc-377186, Santa Cruz Biotechnology). After PBS washing, the slides were incubated with anti-mouse Alexa Fluor 633 (Invitrogen, USA), anti-rat Alexa Fluor 488 (Invitrogen), goat anti-rabbit Alexa Fluor 488 (Invitrogen), goat anti-mouse Alexa Fluor 594 (Invitrogen), goat anti-mouse 488 secondary antibody (Invitrogen), respectively, for 1 h at room temperature. The slides were then washed thrice in PBS, mounted with a fluorescence medium (Vector Laboratories), and analyzed using a fluorescence microscope (Leica Microsystem, Germany).

Five non-overlapping images of each muscle cross-section were taken, and the minimum mouse fiber diameter or cross-sectional area (CSA) was determined using NIS Elements software (Nikon, USA) or Image J software. The minimum ferret diameter was defined as the minimum distance between the opposite boundary of the muscle fiber of parallel tangents and was found to be insensitive to deviation from the optimal cross-sectional profile 1. The data are expressed as percentages. The average TA muscle diameter, fiber CSA threshold for the total 2000–3500 muscle fibers, or GFP intensity was preset in the muscle section using NIS Elements software and remained constant throughout the quantification of all muscles.

### Quantitative RT-PCR

TA muscle tissues were lysed and total RNA was extracted using TRIzol (Invitrogen). Reverse transcription was performed with oligo-dT primers and M-MLV reverse transcriptase (Enzynomics). The resulting cDNA was mixed with TOPreal qPCR 2X PreMIX (SYBR Green with high ROX, Enzynomics) and primers specific to target genes. The qRT-PCR was performed using SYBR Green on an ABI 7500 system. mRNA levels of atrogin-1 (3' AAGCGAAGAGTAAGGCTGTC 5,’ 3' GTGATTGCTTGCAAAGGAAC 5'), MuRF1 (5' TGACCACAGAGGGTAAAG 3,’ 3' TGTCTCACTCATCTCCTTCTTC 5'), MyoD (5' CTTCTATCGCCGCCACTC 3,’ 3' AAGTCGTCTGCTGTCTCAA 5'), MyoG (5' CCAACCCAGGAGATCATTTG 3,’ 3' ACGATGGACGTAAGGGAGTG 5'), MyHC 1 (3' CCAAGGGCCTGAATGAGGAG 5,’ 3' GCAAAGGCTCCAGGTCTGAG 5'), MyHC 2A (3' AAGCGAAGA GTAAGGCTGTC 5,’ 3' GTGATTGCTTGCAAAGGAAC 5'), MyHC 2X (3' CACCGTCTGGATGAGGCTGA 5,’ 3' TGTTTGCGCAGACCCTTGATAG 5'), and MyHC 2B (3' ACAAGCTGCGGGTGAAGAGC 5,’ 3' CAGGACAGTGACAAAGAACG 5') were analyzed.

### Western Blotting

Atrogin-1, MuRF1, MyoD, MyoG, MyHC 1, MyHC 2A, MyHC 2X and MyHC 2B were detected by immunoblotting in separated TA muscle tissue lysate from SGBP, RGX365 administered mice. After sodium dodecyl sulfate (SDS) polyacrylamide gel electrophoresis, immunoblotting assay with mouse anti-atrogin-1 (sc-166806, Santa Cruz Biotechnology), mouse anti-MuRF (sc-398608, Santa Cruz Biotechnology), mouse anti-MyoD (sc-377186, Santa Cruz Biotechnology), mouse anti-MyoG(sc-12732, Santa Cruz Biotechnology), mouse anti- MyHC 1 (BA-D5, DSHB), mouse anti- MyHC 2A (SC-71, DSHB), mouse anti- MyHC 2X (6H1, DSHB), and mouse anti- MyHC 2B (BF-F3, DSHB) were performed.

### Clinical Chemistry and Cytokine Levels in Mouse Plasma

Fresh serum was used to assay alanine aminotransferase (ALT), aspartate aminotransferase (AST), blood urea nitrogen (BUN), creatinine, and lactate dehydrogenase (LDH) using a FUJI DRI-CHEM NX500V at the Chiral Material Core Facility Center of Sungkyunkwan University. To determine the concentrations of IL-1β, IL-6, IL-10, and TNF-α, commercially available ELISA kits were used according to the manufacturer’s protocol (R&D Systems). The values were measured using an ELISA plate reader (Tecan, Austria). Whole blood cell counts were performed using an autohematology analyzer (Mindray, BC 5000 Vet, China) at the Chiral Material Core Facility Center of Sungkyunkwan University.

### Statistical Analysis

All experiments were performed independently at least three times. Data are expressed as mean ± standard deviation (SD). Statistical significance of the differences between the test groups was evaluated using SPSS for Windows (version 16.0; SPSS, USA). Statistical relevance was determined using one-way analysis of variance (ANOVA) and Tukey’s post-hoc test. A value of *p* < 0.05 was considered statistically significant.

## Results

### The Transition from Fresh Ginseng Berry to SGBP Resulted in the Formation of Rare Ginsenosides

The main ginsenosides, such as ginsenosides Rb1, Rc, Rb2, Rd, and Re, contain several sugars, including glucose, rhamnose, and arabinose. Consequently, the polarity and molecular weight of the main ginsenosides are significantly high, resulting in low absorption in the body. Therefore, our research group attempted to research steamed ginseng berries, as they contain many low-molecular weight and low-polarity rare ginsenosides that are well absorbed by the human body. Among the primary components of fresh ginseng berry, the PPT-type ginsenoside Re undergoes structural modifications such as isomerization, hydrolysis, and deglycosylation at the C-20 or C-6 positions. Likewise, the main PPD-type ginsenosides Rb1, Rc, Rb2, and Rd undergo structural transformations such as deglycosylation, isomerization, and hydrolysis at the C-20 position. This resulted in the formation of the following PPT- and PPD-type rare ginsenosides: 20(S)-Rg2, 20(S)-Rh1, 20(R)-Rg2, 20(R)-Rh1, Rg6, Rg4, Rh4, 20(S)-Rg3, 20(R)-Rg3, Rk1, and Rg5 (Fig. 1C). Based on quantification, 1 g of SGBP contained rare ginsenosides in quantities of 4.36, 1.82, 3.24, 1.44, 13.56, 21.24, 9.38, 10.33, 4.59, 33.19, and 37.36 mg, respectively (Table 1).

### High Dosage of SGBP, RGX365 Restored Muscular Strength and Muscle Mass in Aged Mice with Muscle Atrophy

**Fig. 2:**
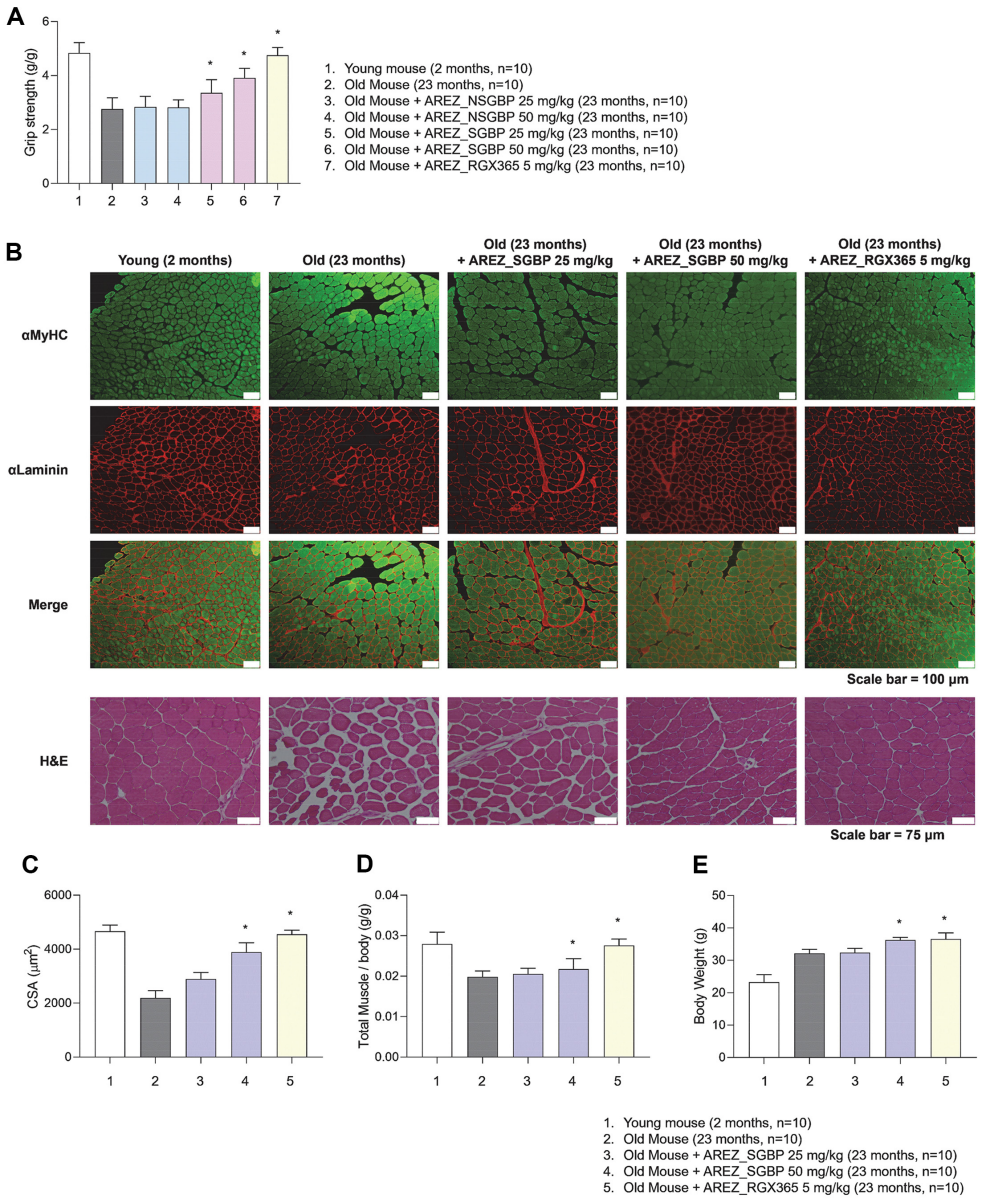
Effect of SGBP and RGX365 on muscle atrophy in aged mice. Two-month-old and twenty-three-month-old male C57BL/6 mice were administered SGBP (25 mg/ml and 50 mg/ml) and RGX365 (5 mg/ml) for 30 days. (**A**) Grip strength. (n=10). (**B**) Immunohistochemistry analyses of MyHC, laminin, and H&E Staining for detection of muscle fiber morphology (scale: 100 μm, 75 μm). (**C**) The mean cross-sectional area (CSA) of each muscle fiber (*n* = 10). (**D**) Total muscle weight per body weight (*n* = 10). (**E**) Body weight of mice at day 30. Data are presented as the mean ± standard deviation (SD); *p < 0.05, **p < 0.01, ***p < 0.001 and ***p < 0.0001 (*t*-test, all experiments were biologically independent). Statistically non-significant results are not indicated in the figure.

To test whether ginseng berry extract could ameliorate naturally developed muscle atrophy in old mice, differently processed extracts were administered at low or high doses for 30 days. The old control group showed reduced grip strength compared with the young control group. The SGBP-administered groups improved significantly, showing a dose-dependent increase; moreover, the RGX365 group recovered grip strength to similar levels as those seen in young mice (Fig. 2A). However, groups treated with non-steamed berry extract (NSGBP) showed no obvious difference, suggesting that only ginseng berries extracted by microwaves under high-pressure or high-temperature conditions exert a surpassing effect on the prevention and treatment of senile sarcopenia.

Next, TA muscle cross-sections were immunostained for MyHC and laminin to check for morphological changes in the muscle fibers. In the aged control group, the muscle fiber cross-sectional size was reduced on account of the degradation of contractile proteins and organelles by activated proteolytic systems [20], resulting in gaps between muscle fibers, indicating muscle atrophy. Groups treated with high-dose SGBP or RGX365 showed significantly more tightly assembled muscle fibers with an increased population and enlarged CSA compared with the aged control group, nearly doubling in size (Fig. 2B and 2C). Consistent with the CSA, the total muscle mass per body weight and body weight was also significantly increased in these groups (Fig. 2D and 2E), underlining the efficacy of SGBP in regenerating weakened muscles caused by senile muscle atrophy.

### High Dosage of SGBP, RGX365 Modulated Ubiquitin-Mediated Protein-Degradation Activity, Facilitating Muscle Differentiation

**Fig. 3:**
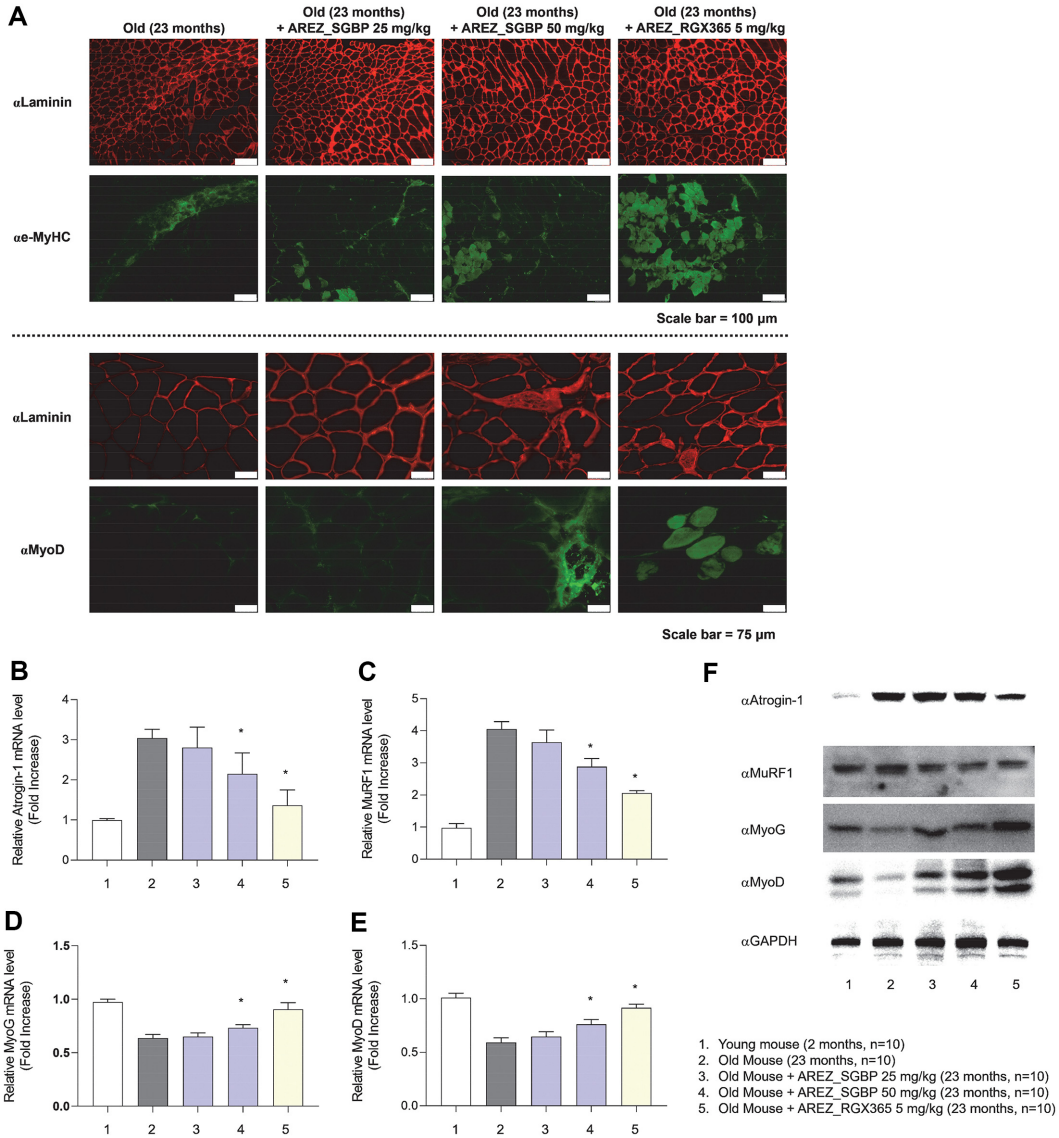
SGBP and RGX365 facilitate myogenesis and suppress muscle degradation mechanisms. Immunohistology of muscle cross-sections was used to confirm muscle regeneration in TA muscle tissue. (**A**) Immunohistochemistry analyses of laminin, embryonic MyHC, and MyoD for detection of myogenesis (scale: 75 μm). (**B, C**) Analysis of relative mRNA expression of factors related to muscle degradation (atrogin-1, MuRF1) and (**D, E**) muscle myogenesis (MyoG, MyoD) (*n* = 10).) (**F**) Western blot data related myogenesis and muscle degradation influenced by SGBP and RGX365. Data are presented as the mean ± standard deviation (SD); *p < 0.05, **p < 0.01, ***p < 0.001 and ***p < 0.0001 (*t*-test, all experiments were biologically independent). Statistically non-significant results are not indicated in the figure.

To further investigate the potential effects of SGBP and RGX365 on muscle regeneration, immunostaining was performed on TA muscle cross-sections. Embryonic MyHC (e-MyHC) [21], known to be upregulated in immature myofibers during muscle regeneration, and MyoD [22], a key transcription factor involved in muscle differentiation, were used as markers. Groups that received SGBP or RGX365 showed increased expression of e-MyHC and MyoD, confirming their role in muscle repair (Fig. 3A).

Based on these findings, the mRNA and protein expression levels of E3 ubiquitin ligases, known to contribute to sarcopenia (atrogin-1 and MuRF1) [23], as well as the family of myogenic regulatory factors (MRFs) (MyoD and MyoG) [22], were assessed in TA muscles. Mice administered with high doses of SGBP and RGX365 exhibited a significant reduction in the expressions of atrogin-1 and MuRF1, indicating the activation of myogenesis and suppression of muscle protein degradation associated with aging (Fig. 3A, 3B, and 3F). Moreover, the expression of MyoD and MyoG was upregulated (Fig. 3D-3F). Administering high doses of SGBP and RGX365 effectively regulated the abnormal expression of MRFs in aged mice, thereby promoting myogenesis and muscle regeneration.

### SGBP and RGX365 Suppressed NF-κB Activation and Serum Cytokine Levels in Aged Mice

The inflammatory response is a crucial factor in the progression of aging-induced sarcopenia [24]. As the concentration of cytokines in the bloodstream rises and NF-κB activation occurs, the expression of proteins associated with sarcopenia is upregulated as well [25-27]. Therefore, we aimed to investigate the potential of SGBP and RGX365 in mitigating the inflammatory response in aged mice.

**Fig. 4:**
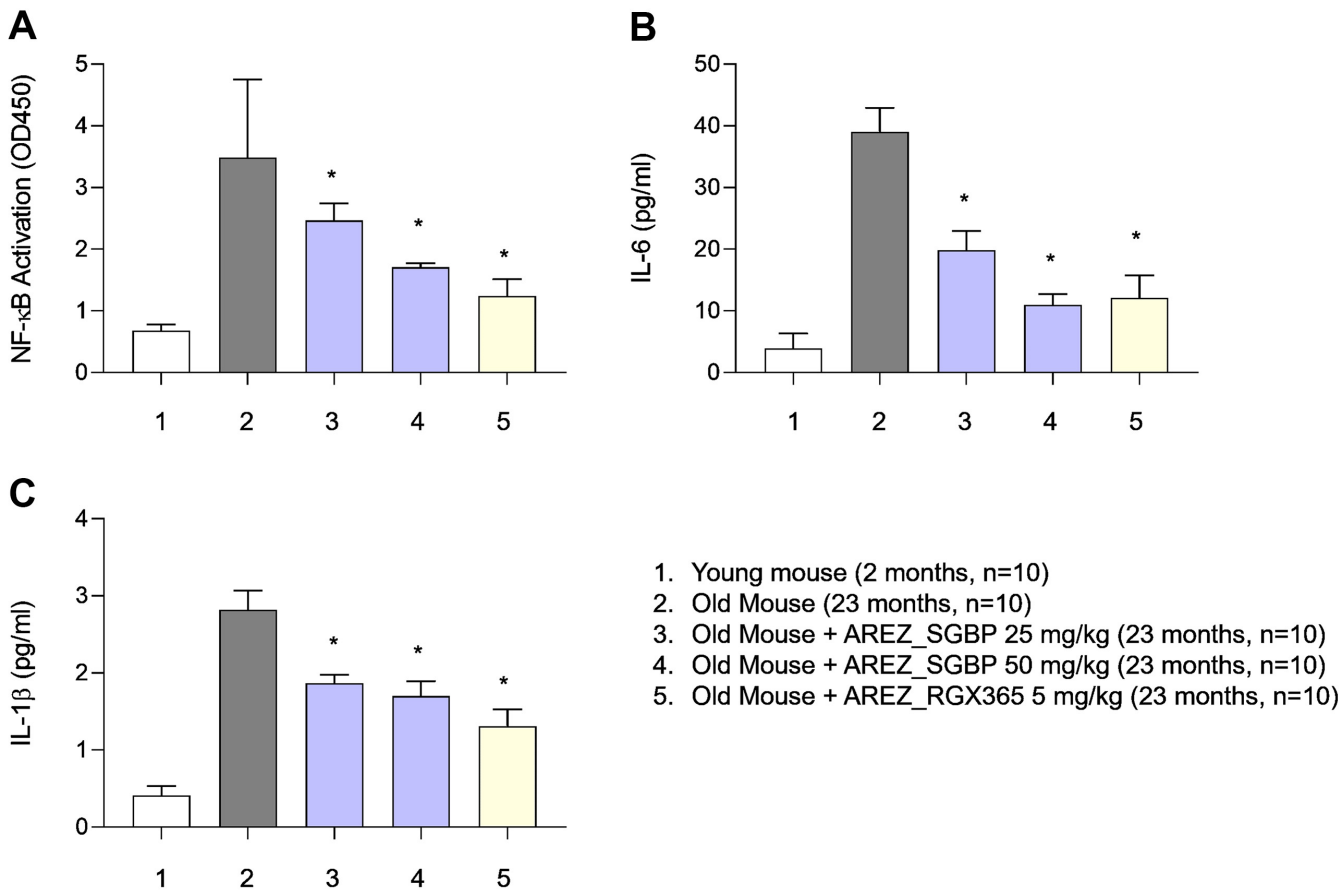
Effect of SGBP and RGX365 on NF-κB activation and serum cytokine levels in aged mice. ELISAs of changes in (**A**) NF-κB activation, (**B**) IL-6 level, (**C**) IL-1β level in the blood in each group (*n* = 10). Data are presented as the mean ± standard deviation (SD); *p < 0.05, **p < 0.01, ***p < 0.001 and ***p < 0.0001 (*t*-test, all experiments were biologically independent). Statistically non-significant results are not indicated in the figure.

To this end, we assessed NF-κB activation in muscle tissues and quantified the levels of proinflammatory cytokines in the blood. Notably, we observed a significant dose-dependent decrease in NF-κB levels in the muscle tissue of mice treated with SGBP compared with the corresponding NF-κB levels in the control group (Fig. 4A). Consequently, the levels of IL-6 and IL-1β in the bloodstream were notably reduced (Fig. 4B and 4C). Similarly, RGX365 also demonstrated the capacity to lower NF-κB levels in muscle tissue, leading to decreased IL-6 and IL-1β levels in the blood (Fig. 4A-4C). Notably, among all the groups studied, RGX365 exhibited the most effective reduction in the inflammatory response (Fig. 4A-4C). Our findings highlighted the ability of SGBP and RGX365 to effectively suppress the inflammatory response in mice with sarcopenia. Notably, RGX365 demonstrated the highest efficacy in reducing the levels of inflammatory markers. These results offered promising insights into the potential use of SGBP and RGX365 as interventions to effectively mitigate the inflammatory processes associated with sarcopenia.

### SGBP and RGX365 Promoted Myogenesis by Enhancing MyHC Expression

**Fig. 5:**
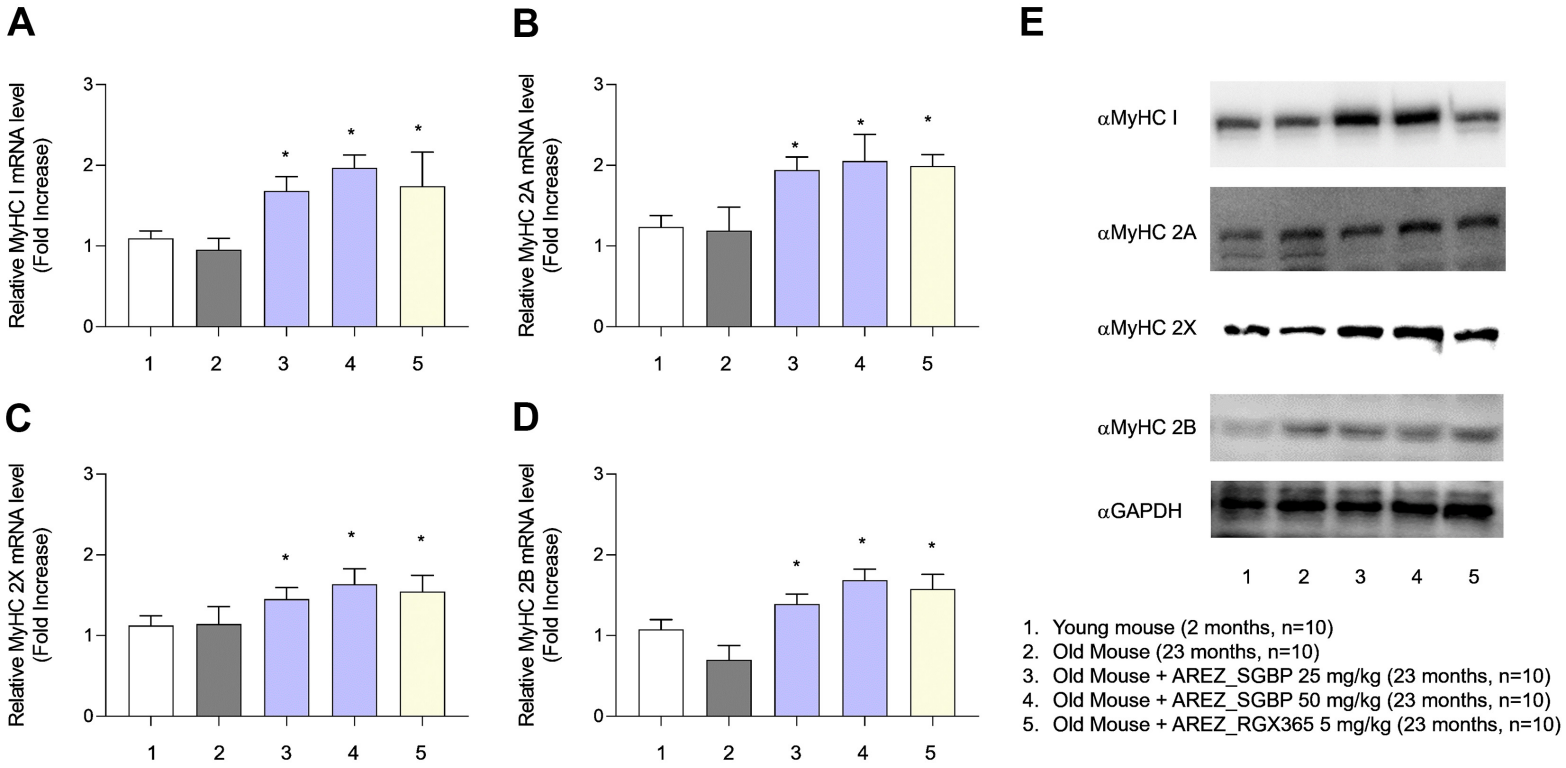
SGBP and RGX365 promote muscle regeneration by improvement in MyHC mRNA expression. Changes in mRNA levels in four different isomers of MyHC in the TA muscle of SGBP-treated mice. The MyHC isomers include (**A**) MyHC 1, (**B**) MyHC 2A, (**C**) MyHC 2X, and (**D**) MyHC 2B (n=10). (**E**) Protein expression levels of MyHC isomers were evaluated with western blot analysis. Data are presented as the mean ± standard deviation (SD); *p < 0.05, **p < 0.01, ***p < 0.001 and ***p < 0.0001 (*t*-test, all experiments were biologically independent). Statistically non-significant results are not indicated in the figure.

MyHC is a major component of contractile proteins that act as motor proteins in the muscle tissue. Therefore, we investigated whether SGBP and RGX365 facilitate MyHC expression. We analyzed four MyHC isoforms (MyHC1, MyHC2A, MyHC 2X, and MyHC 2 B). SGBP and RGX365 significantly upregulated the mRNA level of all MyHC isoforms (MyHC1, MyHC2A, MyHC 2X, and MyHC 2B) (Fig. 5A-5D). Protein levels of MyHC isomers were also elevated in the TA muscle of SGBP and RGX365 administered mice. These results suggested that SGBP ameliorates sarcopenia through the following mechanisms: alleviation of the inflammatory response and improvement of myogenesis (Figs. 4 and 5).

MyHC is a pivotal constituent within the realm of contractile proteins and serves as an indispensable motor protein within the intricate fabric of muscle tissue. This pivotal role motivated us to comprehensively explore whether the administration of SGBP and RGX365 could effectively facilitate the amplification of MyHC expression. Our investigative studies included a meticulous analysis of the following four distinctive MyHC isoforms: MyHC1, MyHC2A, MyHC 2X, and MyHC 2B. The obtained findings revealed a robust pattern of upregulation across the entire spectrum of MyHC isoforms, which was consistently observed in both the SGBP-and RGX365-treated groups (Fig. 5).

These findings shed light on the potential role of SGBP in alleviating sarcopenia through a multifaceted approach. By dampening the inflammatory response (Fig. 4) as well as fostering myogenesis and muscle regeneration (Fig. 5), SGBP demonstrates promising therapeutic effects.

### Sub-Chronic Oral Toxicity

**Fig. 6:**
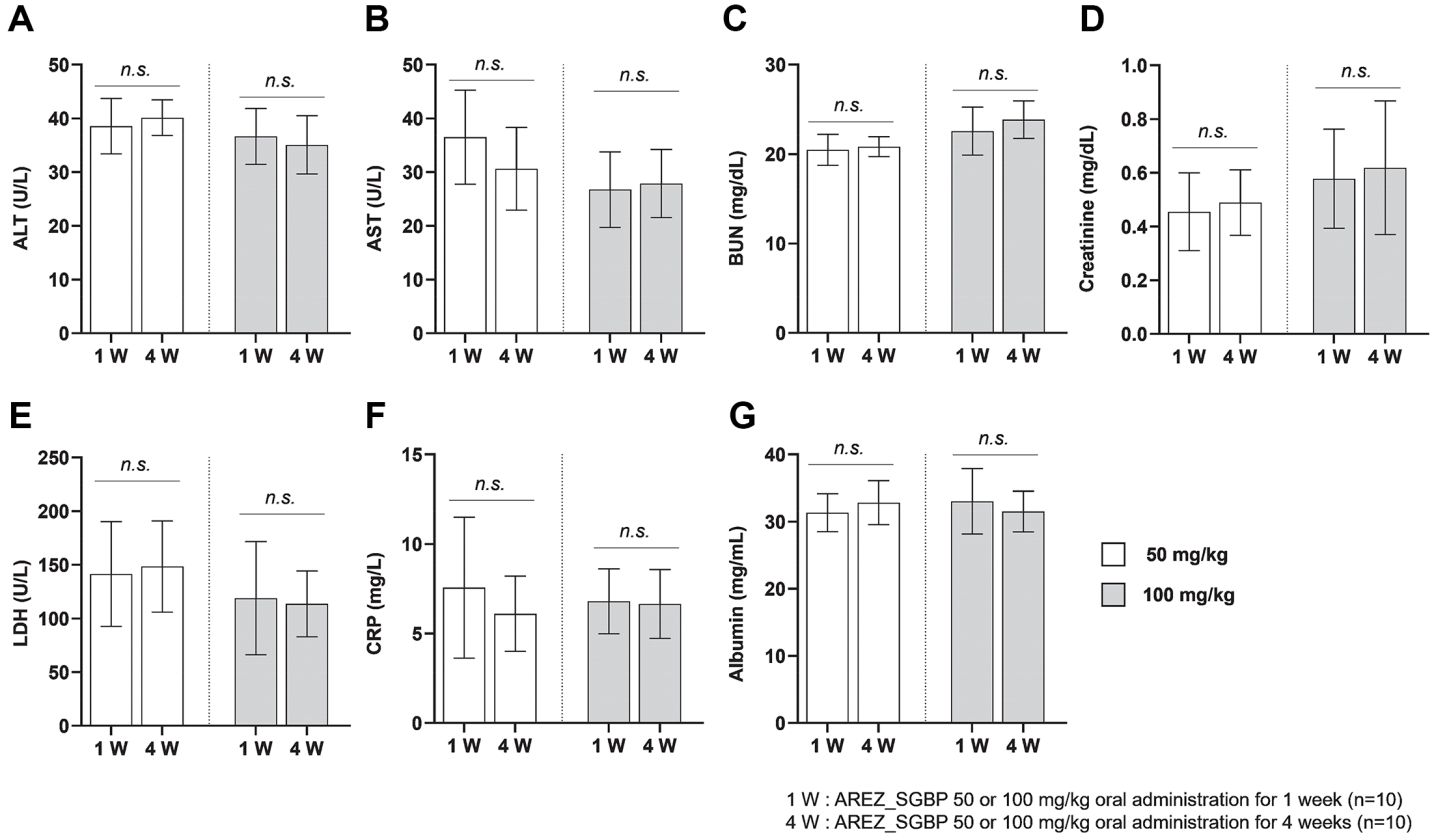
Non-toxic effect of SGBP on various organs and the immune system. All markers have been measured in C57BL/6 mice treated with SGBP 50 mg/kg or 100 mg/kg oral administration for 1 week or 4 weeks. (**A-D**) Analysis of organdamage markers in the blood of SGBP-treated mice. The markers include ALT, AST, BUN, and creatinine (*n* = 10). (**E, F**) Analysis of immune-activation markers in the blood of SGBP-treated mice. (**G**) Analysis of albumin levels of SGBP-treated mice. Data are presented as the mean ± standard deviation (SD); *p < 0.05, **p < 0.01, ***p < 0.001, and ***p < 0.0001 (*t*-test, all experiments were biologically independent). Statistically non-significant results are not indicated in the figure.

No indications of external toxicity were observed in either the treatment or vehicle control groups during the 28-day administration period. The animals displayed normal behavior and physical condition, and no significant abnormalities in clinical signs were noted throughout the study. The hematological and serum biochemical data from the 29-day study are summarized in Fig. 6.

To evaluate the potential drug toxicity mechanisms, we conducted blood biochemical assays targeting specific tissues, particularly the kidneys and liver. Notably, there were no significant differences in the hematological data when compared to the normal ranges. Additionally, we explored whether SGBP influenced the production of proinflammatory cytokines (Fig. S1) and blood cell ratio (Fig. S2). This indirect assessment affirmed the safety of SGBP within the body, even during extended periods, akin to the longstanding use of Korean ginseng.

This paragraph underscores the absence of adverse effects or toxicity and provides insights into the safety profile of SGBP through various analyses, offering a parallel to the well-established safety of long-term Korean ginseng consumption.

## Discussion

Finding the optimal parameters for the manufacturing process of natural product drugs is important for drug development to maximize efficacy and obtain a good yield of byproducts. Major PPD- and PPT-type ginsenosides, such as ginsenosides Rb1, Rb2, Rc, Rd, and Re, are highly polar because of the presence of several sugar groups with high molecular weights. These structural features of the main ginsenosides result in poor bioavailability and absorption efficiency in the body owing to hydrolysis and hepatic metabolism [28, 29].

Appropriate steaming conditions and the number of iterations of the steam-drying process result in deglycosylation of the main ginsenosides, changing them to a suitable structure amenable to higher absorption and well-established anti-inflammatory and immunomodulatory effects [30]. Unlike active investigations dedicated to the root of *Panax ginseng* roots, ginseng berry had been overlooked until recent research revealed its higher ginsenoside content than that in the roots. The major consumption groups of *Panax ginseng* byproducts are elderly individuals with sarcopenia. Treatment of senile sarcopenia is difficult because physical exercise for rehabilitation for the elderly may be quite underwhelming, thus leaving no alternative other than a pharmacological approach. Therefore, we hypothesized that ginseng berry extract manufactured through a steam-drying process could ameliorate senile sarcopenia.

In comparison with SGBP, NSGBP-administered mice did not regain muscle strength irrespective of dosage, indicating the importance of processing ginsenosides into rare ginsenosides by steaming. Cross-sections of SGBP-administered mice showed recovered myotube formation, and double immunostaining for embryonic MyHC or MyoD with laminin showed increased fluorescent regions, suggesting the restoration of myogenesis. In parallel, the mRNA expression of MyoG and MyoD was significantly upregulated, whereas sarcopenia inducing E3 ubiquitin ligases that degrade muscle proteins were repressed.

The NF-κB transcription factor family is known to play a key role in inflammatory pathways. In aged people, proinflammatory cytokines such as IL-1β, and IL-6 transcribed by NF-κB can be aberrantly accumulated, breaking the balance of the oxidation–reduction status of the cells, and inducing skeletal muscle atrophy [25-27].

As expected, SGBP raised blood cytokines levels and mediated increased NF-κB activation, confirming its anti-inflammatory efficacy. Furthermore, augmented mRNA levels of the four different MyHC isoforms may facilitate myogenesis owing to regulated inflammatory responses in senile sarcopenia. Additionally, the safety of SGBP application was verified by analyzing abnormal levels or dramatic changes in organ damage markers, blood cytokine levels, and blood cell damage after short- and long-term administration.

In summary, we investigated whether administration of SGBP for 30 days could effectively alleviate senile sarcopenia in aged mice by evaluating the improvement in muscle morphology and physical ability, repression of sarcopenia-inducing molecule expression, and toxicity tests. Although SGBP did not show better efficacy than the previously developed RGX365, a high dosage of SGBP still showed excellent therapeutic effects in senile sarcopenia mouse models, delineating the prospect of clinical application.

## Supplemental Materials

Supplementary data for this paper are available on-line only at http://jmb.or.kr.



## Figures and Tables

**Table 1:** The amount of 1 g of steamed ginseng berry extract (SGBP).

Rare ginsenoside (PPT)	Amount (mg)	Rare ginsenoside (PPD)	Amount (mg)
20(S)-Rg2	4.36	20(S)-Rg3	10.33
20(S)-Rh1	1.82	20(R)-Rg3	4.59
20(R)-Rg2	3.24	Rk1	33.19
20(R)-Rh1	1.44	Rg5	37.36
Rg6	13.56		
Rg4	21.24		
Rh4	9.38		
