# Supplementary material for: Steamed Ginseng Berry Powder Ameliorates Skeletal Muscle Atrophy via Myogenic Effects
Source: J Microbiol Biotechnol. 2023 Nov 17;34(1):157–66. doi: 10.4014/jmb.2309.09017 (PMC10840479; doi:10.4014/jmb.2309.09017)
Supplement: Supplementary file 1 [file jmb-34-1-157-supple.pdf]

J. Microbiol. Biotechnol.
https://doi.org/10.4014/jmb.2309.09017

# Supplementary Figures

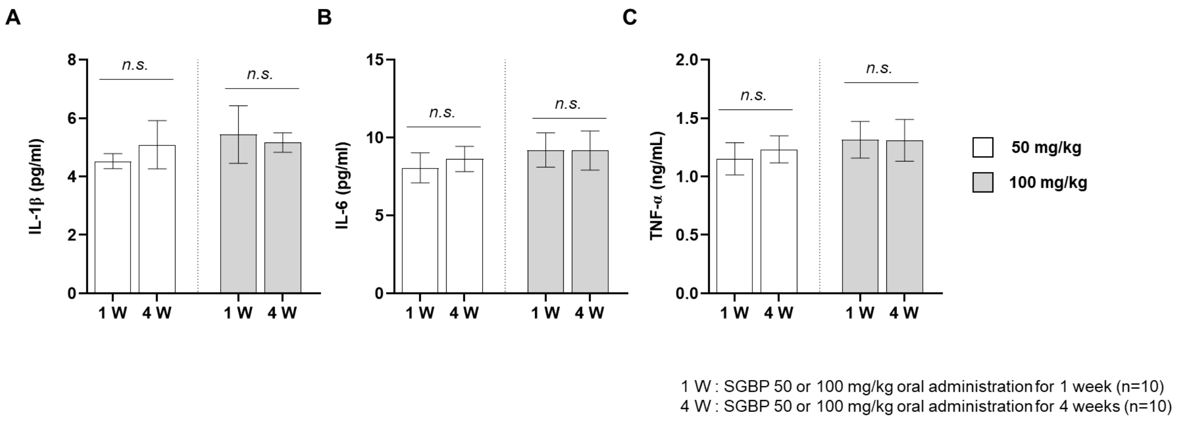


**Fig. S1. No increase in blood cytokine levels was observed in mice exposed to steamed ginseng berry extract, irrespective of the concentration or exposure time.** (**A-C**) Changes in blood cytokine levels in mice after treatment with steamed ginseng berry extract. The cytokines include IL-1β, IL-6, and TNF-α (n = 10). All markers were measured in C57BL/6 mice treated with SGBP 50 mg/kg or 100 mg/kg oral administration for 1 week or 4 weeks.

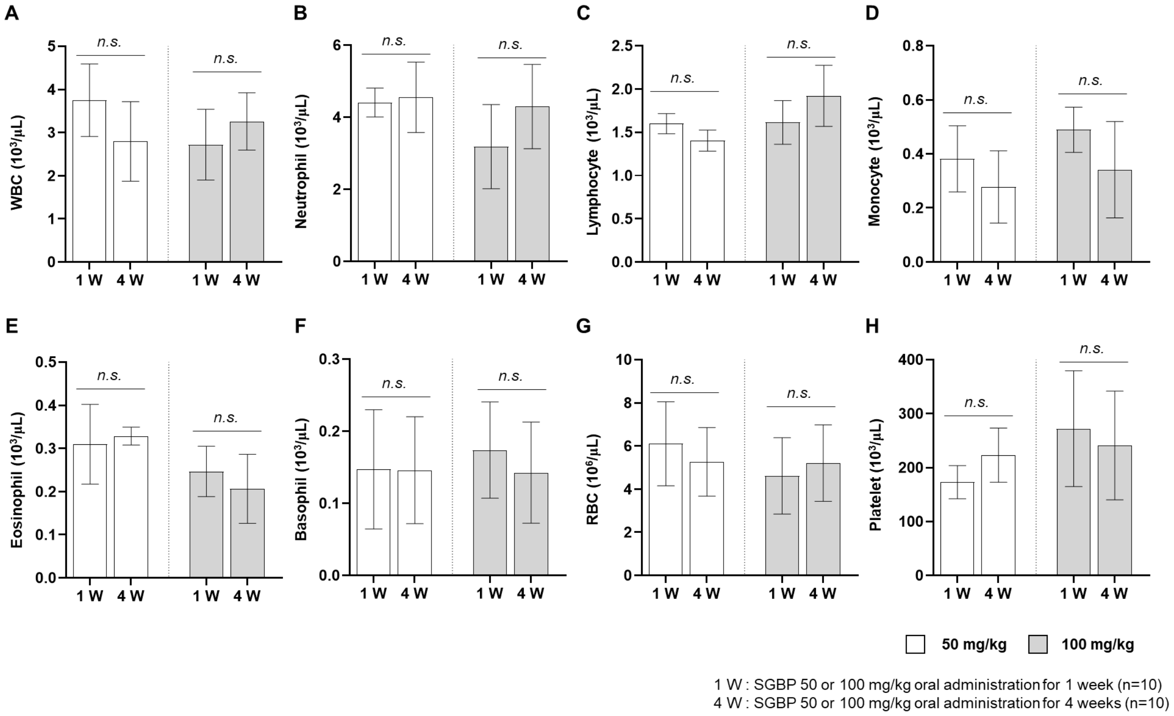


**Fig. S2. No effect of steamed ginseng berry extract on blood cell damage was observed, irrespective of the concentration and exposure time.** (**A-G**) Quantification of mean blood cells in steamed ginseng berry extract-treated mice. Cell counts (cells × $10^3$/μL) are presented as WBCs, neutrophils, lymphocytes, monocytes, eosinophils, basophils, and RBCs in each group (n=10). (**H**) Platelet count after the administration of steamed ginseng berry extract. All markers were measured in C57BL/6 mice treated with SGBP 50 mg/kg or 100 mg/kg oral administration for 1 week or 4 weeks.
